# Fragmentation Dynamics of Benzoyl Peroxide: Insights from Rotational Spectroscopy

**DOI:** 10.1021/acs.jpclett.5c02600

**Published:** 2025-10-30

**Authors:** Sergio Mato, Sofía Municio, José Luis Alonso, Elena R. Alonso, Iker León

**Affiliations:** † Grupo de Espectrocopía Molecular (GEM), Edificio Quifima, Laboratorios de Espectroscopia y Bioespectroscopia, Unidad Asociada CSIC, Parque Científico UVa, 16782 Universidad de Valladolid, 47011 Valladolid, Spain

## Abstract

Benzoyl peroxide (BPO) represents a structurally simple yet hazardous organic peroxide with widespread applications across industrial and pharmaceutical domains. Despite its extensive use, detailed molecular-level understanding of its thermal instability remains limited. Here, we present the first rotational spectroscopy characterization of isolated BPO in the gas phase, enabled by laser ablation and supersonic jet expansion techniques. Our analysis reveals a *C*_2_-symmetric structure with quasi-perpendicular aromatic rings, in excellent agreement with crystallographic data. Quantum chemical calculations and topological analysis identify a stabilizing reciprocal n→π* interaction between adjacent carbonyl groups that may contribute to BPO’s thermal resilience compared to other organic peroxides. Furthermore, we detect several photofragmentation products, including benzoic acid, benzyne, benzaldehyde, and benzophenone, providing insights into potential decomposition pathways. This molecular-level investigation bridges the gap between macroscopic hazard assessments and fundamental understanding of peroxide reactivity, with implications for safer handling and rational design of peroxide-based systems.

## Graphical abstract

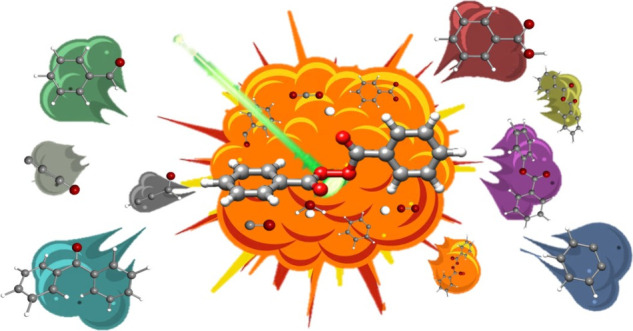

Organic peroxides (R–O–O–R′) represent a structurally simple yet functionally diverse class of compounds that play pivotal roles across atmospheric, synthetic, industrial, and biological contexts. Their propensity to undergo homolytic O–O bond cleavage under mild conditions makes them invaluable as radical initiators, particularly in polymerization reactions and oxidative transformations.^1–3^ Beyond synthetic utility, organic peroxides are endogenously produced and metabolized in living systems, where they exhibit a range of biological activities and have garnered interest as pharmacological agents.^4^ However, the intrinsic weakness of the peroxide linkage (−O–O−) imparts considerable thermal lability and a pronounced risk of runaway decomposition, rendering these compounds inherently hazardous.^5^ A nuanced understanding of the structure–reactivity relationships governing peroxide stability is therefore critical to both their safe handling and the rational design of functional peroxide-based systems.

Among the array of organic peroxides, benzoyl peroxide (BPO) occupies a central position as a prototypical and widely deployed member of this family. Its utility spans from industrial-scale polymer production, where it functions as a key initiator, to dermatological formulations for acne treatment.^6–8^ Despite its widespread application, BPO is classified as a highly hazardous substance due to its thermal instability, which has been implicated in numerous industrial incidents. These events underscore the urgent need to elucidate the mechanistic pathways governing its decomposition. The primary thermal decomposition of BPO proceeds via homolytic cleavage of the O–O bond, generating benzoate radicals, as shown in Scheme 1.

**Scheme 1:**
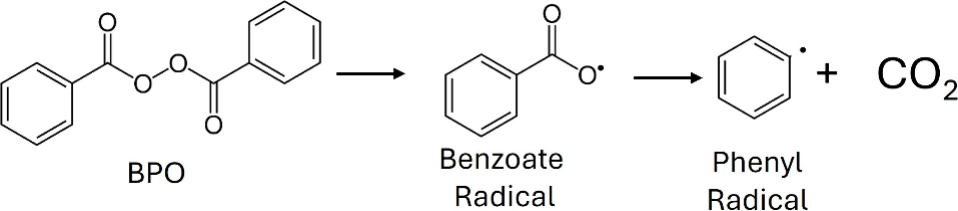
Main BPO Decomposition Reaction Showing the Homolytic Cleavage of the O–O Bond to Form Benzoate Radicals

Extensive investigations have employed kinetic modeling and calorimetric analyses to quantify BPO’s decomposition parameters, including activation energies, reaction enthalpies, and temperature-dependent rate constants.^9–19^ While these macroscopic studies have laid the foundation for regulatory guidelines and hazard assessments, they fall short in capturing the fundamental molecular-level processes that underline peroxide reactivity and instability.

Herein, we propose a molecular-level investigation of bare BPO via rotational spectroscopy in the gas phase, enabled by supersonic jet expansion and laser ablation methods, complemented by high-level quantum chemical calculations. This strategy allows for an unambiguous characterization of BPO’s conformational landscape, intramolecular interactions, and potential energy surfaces with unmatched resolution.^20,21^ Notably, the plasma environment generated during laser ablation promotes molecular fragmentation and recombination which,^22,23^ together with the isolation conditions of the supersonic expansion, facilitates access to transient species and short-lived intermediates otherwise inaccessible under thermal conditions. By probing these elusive species, our approach seeks to uncover mechanistic pathways that contribute to the thermal lability and explosive potential of BPO. Ultimately, this work aims to bridge the gap between macroscopic hazard assessments and the fundamental molecular dynamics that govern peroxide decomposition.

As a starting point, a comprehensive conformational search of BPO was performed, as described in the Computational Methods section. The analysis yielded three distinct conformers (see Figure 1 and Table 1, as well as Figure S1 and Table S1), with one conformer significantly more stable than the others. As shown in Figure 1, the conformers differ from each other in the relative position of the aromatic rings: The most stable structure, BPO-I, has the two rings adopting a quasi-perpendicular orientation in an extended molecular conformation. The most unstable structures obtained, BPO-II and BPO-III, adopt a non-extended configuration decreasing their stability by approximately 2000 cm^–1^ respect to the global minimum. According to the energetics, only the global minimum is expected to be populated under the conditions of a supersonic jet expansion. Additionally, the global minimum exhibits *C*_*2*_ symmetry (Figure 1), and the calculated Ray’s asymmetry parameter (κ ≈ −0.997) confirms a nearly prolate symmetric top geometry. The dipole moment is oriented exclusively along the *c*-axis, which should make *c*-type rotational transitions the dominant features in the spectrum.

**Table 1:** Comparison between the Theoretical Spectroscopic Constants for the Three Conformers of BPO and the Experimental Rotational Constants of the Detected Rotamer

	Experimental	Calculated		
	Rotamer 1	I	II	III
*A* ^a^	1708.96035(102)^g^	1706	693	694
*B*	163.616287(244)	163	290	276
*C*	162.368382(283)	161	247	231
|μ_*a*_|	Not observed	0.0	3.9	3.2
|μ_*b*_|	Not observed	0.0	5.2	5.7
|μ_*c*_|	Observed	1.1	1.2	0.8
σ^b^	27.9			
N^c^	69			
ΔE^d^		0	1685	2122
ΔE_ZPE_ ^e^		0	1614	2037
ΔG^f^		0	1819	2083

a *A, B*, and *C* represent the rotational constants (in MHz); μ_*a*_, μ_*b*_, and μ_*c*_ are the electric dipole moment components (in D).

b RMS deviation of the fit (in kHz).

c Number of measured transitions.

d Relative energies (in cm^–1^) with respect to the global minimum.

e Relative energies (in cm^–1^) considering the zero-point energy (ZPE).

f Gibbs energies (in cm^–1^) calculated at 298 K and 1 atm.

g Standard error in parentheses in units of the last digit.

**Figure 1:**
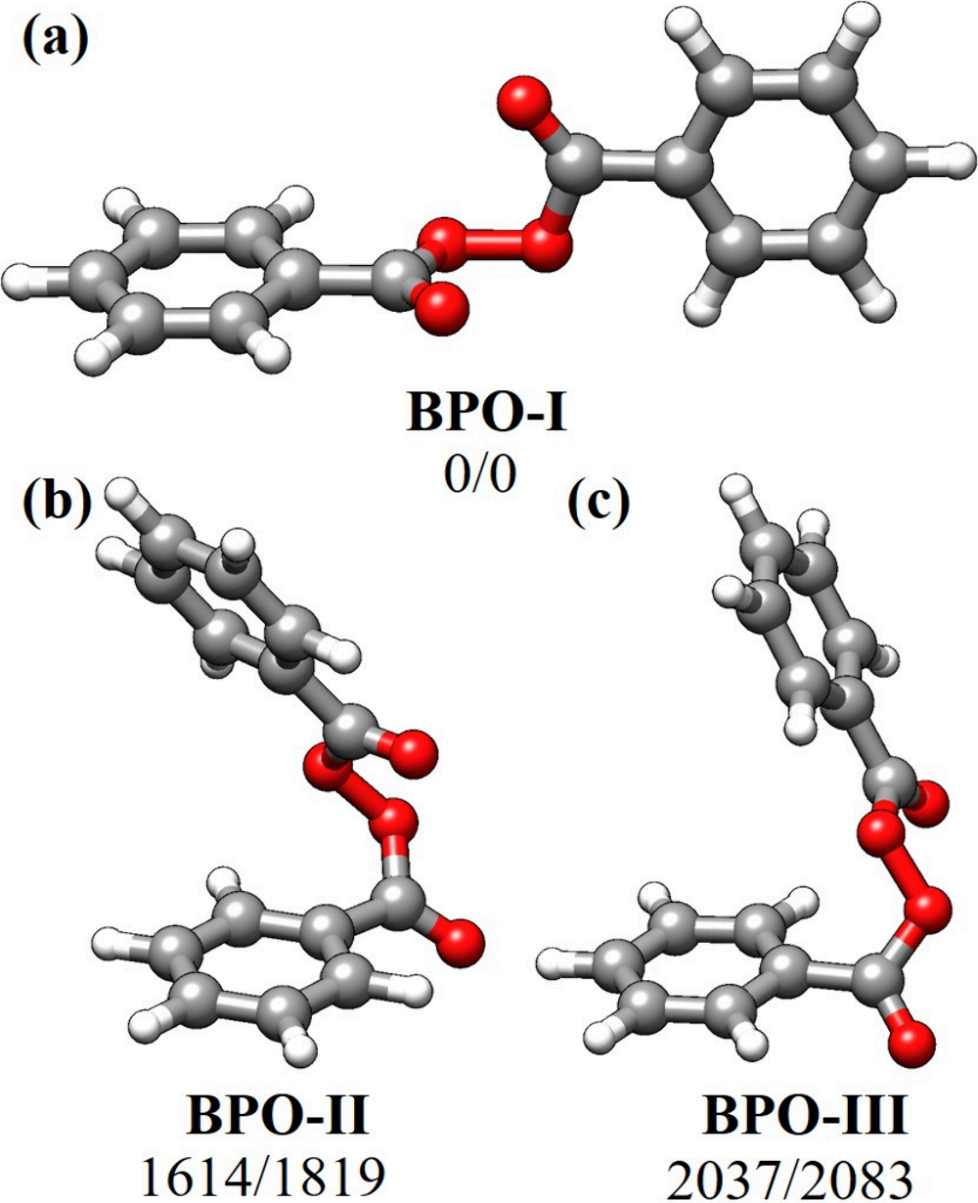
Low-lying conformations of BPO. (a) BPO-I; (b) BPO-II; (c) BPO-III. The bottom shows the energy difference considering the zero-point energy correction (ΔE_ZPE_), as well as the entropic difference at room temperature and 1 bar (ΔG) in the ΔE_ZPE_/ΔG form (in cm^–1^).

The rotational spectrum of BPO was recorded using our laser ablation chirped-pulse Fourier transform microwave (LA-CP-FTMW) spectrometer operating in the 6–14 GHz frequency range (Figure 2).^24–26^ The use of laser ablation followed by supersonic jet expansion is particularly advantageous for thermolabile compounds such as BPO because it provides rapid vaporization followed by immediate cooling, preventing thermal decomposition that would occur during conventional heating methods. BPO decomposes at temperatures above 378 K, making laser ablation the method of choice for allowing their characterization in the gas phase.

**Figure 2:**
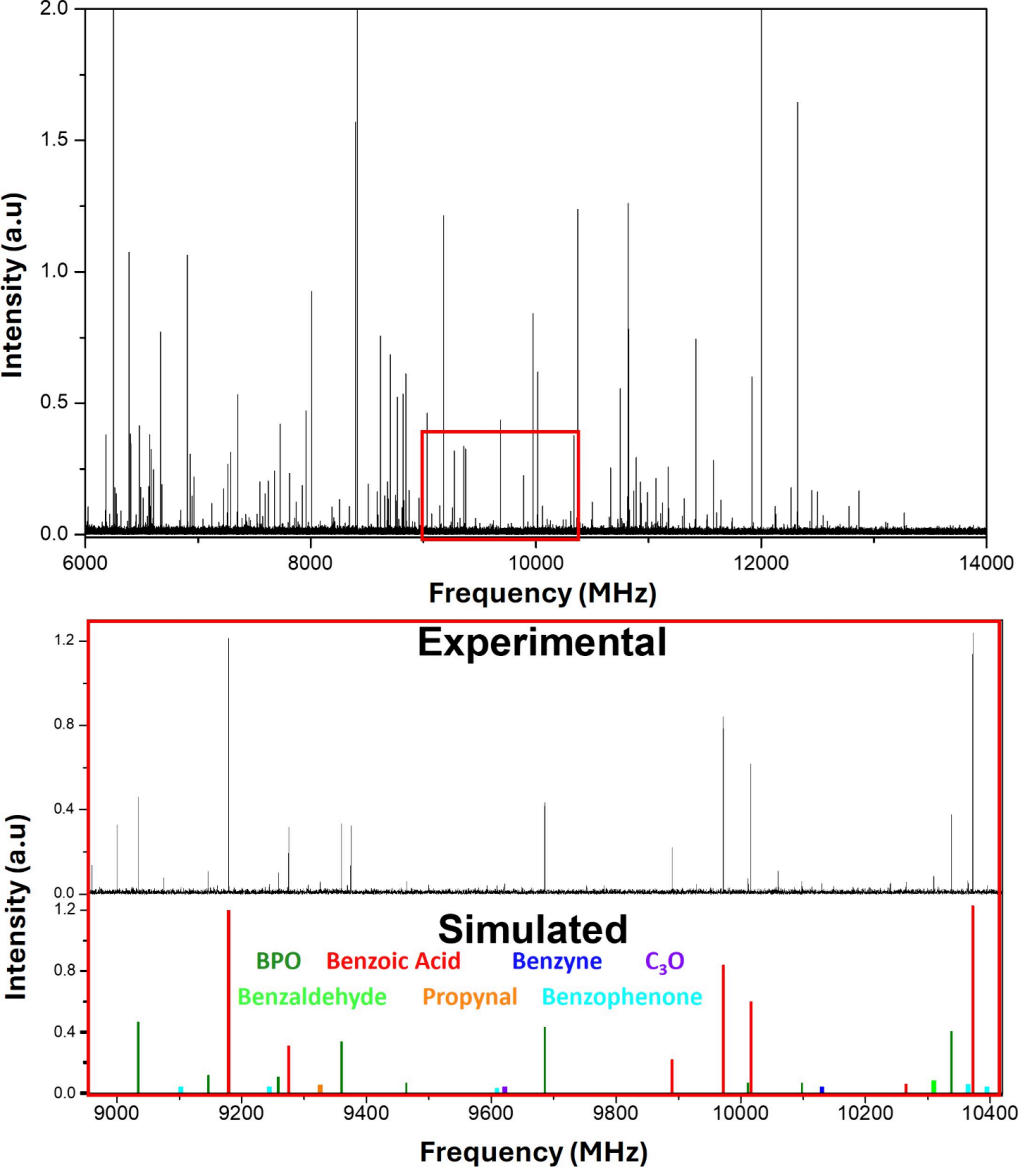
(Top) Experimental rotational spectrum of BPO in the 6–14 GHz frequency range. (Bottom) Zoomed spectrum from 9 to 10.5 GHz comparing some selected rotational lines of BPO and its fragments.

Guided by theoretical predictions, we began the spectral assignment by searching for *R*-branch *c*-type transitions and identified a well-defined pattern of lines separated by approximately ∼ 330 MHz (2B), which could be attributed to a first rotamer. Using a standard rigid rotor model,^27–31^ we measured and fitted a total of 69 rotational transitions, yielding highly accurate rotational constants summarized in Table 1. The frequency transitions are collected in Table S1.

After subtracting these assigned transitions from the experimental spectrum, several residual lines remained. Despite an exhaustive search and comparison with calculated spectra for potential BPO-related species or its water clusters, no additional rotamers could be identified at this stage, in agreement with the high instability of any other conformers. These residual signals are thus tentatively attributed to photofragmentation products generated during laser ablation and will be discussed below.

The final step in the analysis involved unambiguous conformational identification. The experimentally determined rotational constants provide direct information on the mass distribution and geometry of the observed species. A comparison between the determined values and those predicted in Table 1 reveals excellent agreement for the lowest-energy structure (BPO-I). The rotational constants match within a scaling factor of 1.002 to 1.011, strongly indicating that the theoretical geometry closely reproduces the actual gas-phase structure. Additionally, only *c*-type transitions were observed in the rotational spectrum in good agreement with the dipole moments.

Once the conformational assignment was completed, we sought to understand the implications of the gas-phase structure on the compound’s physicochemical behavior. To this end, we compared our experimentally derived gas-phase geometry with the reported crystal structure. As shown in Figure 3, both structures exhibit excellent agreement. This strong structural correspondence suggests that the intrinsic molecular conformation of BPO is largely preserved across phases. Consequently, our gas-phase data can be reasonably extrapolated to interpret aspects of the compound’s behavior in the condensed phase which, we anticipate, its fragile stability is due to n→π* interaction between the carbonyl groups. This finding supports the broader relevance of gas-phase structural studies in understanding the fundamental stability and reactivity of molecules, including peroxide systems under various conditions.

**Figure 3:**
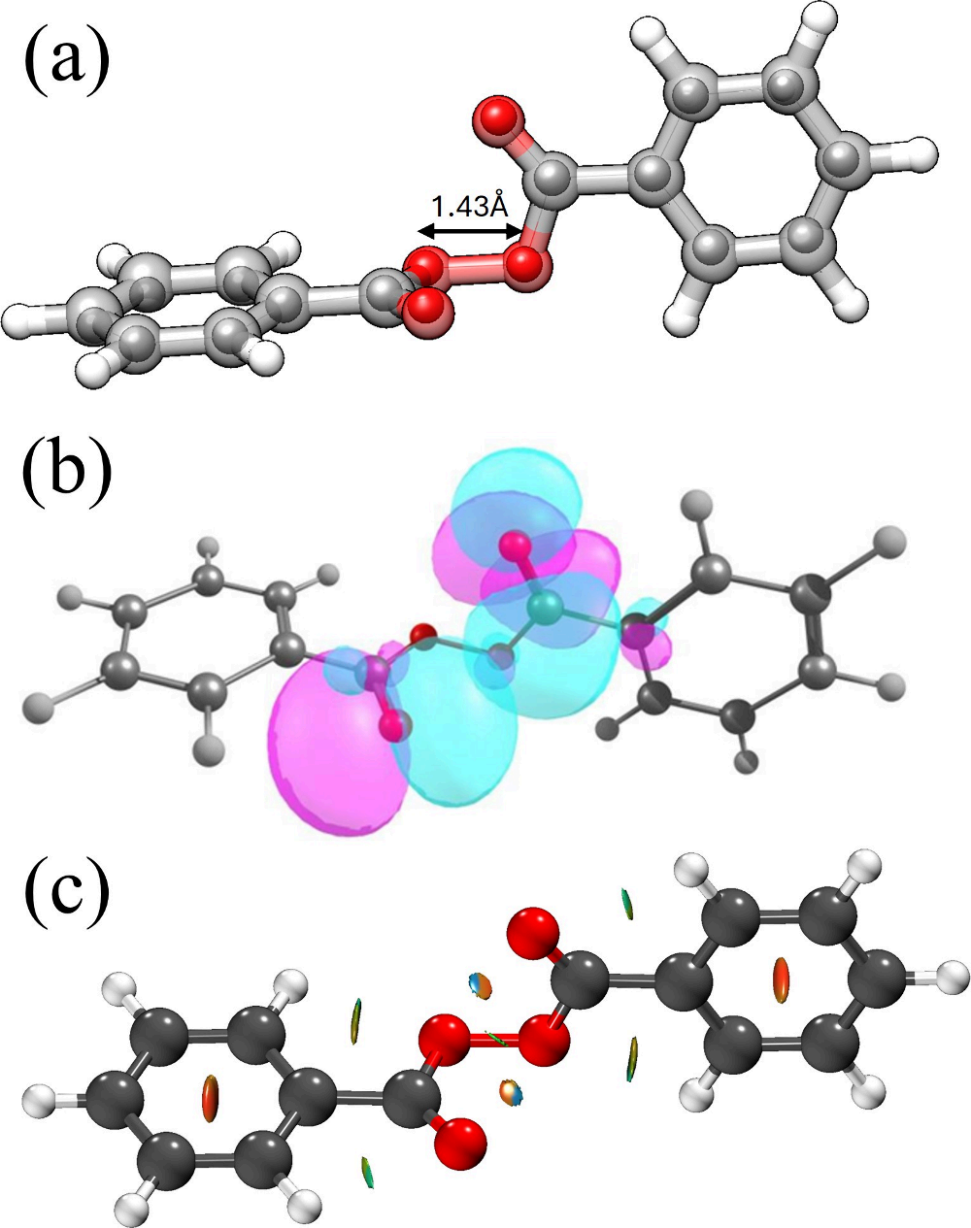
(a) Overlay of the gas phase structure of BPO and the crystal structure (inner spheres).^32^ The coordinates from crystal BPO were taken from the provided structure from the Cambridge Crystallographic Data Centre (CCDC) webpage. (b) NBO representation of n→π* interaction. (c) NCIplot representation of noncovalent interactions. Red surfaces correspond to strong repulsion forces, blue surfaces to strong attraction forces and green surfaces to weak attractive interactions. An isovalue of 0.35 a.u was used.

To gain deeper insight into the factors governing the stability of the observed conformation, we investigated the intramolecular interactions that may contribute to the molecular architecture of BPO. For this purpose, we performed a detailed topological analysis using three complementary approaches: Natural Bond Orbital (NBO),^33^ Quantum Theory of Atoms in Molecules (QTAIM, see Table S3),^34^ and Non-Covalent Interaction (NCI, see Figure S2).^35,36^ As can be seen in Figure 3, BPO-I is stabilized by four C–H···O intramolecular interactions. More importantly, the analyses revealed a dominant reciprocal n→π* interaction involving a short contact between adjacent carbonyl groups (C=O···C=O), with a stabilization energy of 0.27 kcal mol^–1^ as estimated by second-order NBO perturbation theory. Although modest in absolute magnitude, this interaction may contribute to the conformational rigidity of BPO and could represent one of several factors that differentiate BPO from other organic peroxides lacking such stabilizing interactions by reinforcing the O–O bond and thermal resilience of BPO.

This type of electron delocalization is not limited to small organic molecules, it plays a well-established structural role in biomolecular systems, particularly in the stabilization of protein secondary and tertiary structures.^37–48^ The identification of this interaction in BPO thus provides a compelling molecular analogy, suggesting that similar delocalization mechanisms may underpin conformational preferences and reactivity differences not only among peroxides, but also across broader chemical and biological frameworks where weak, yet highly directional, intramolecular forces govern structural integrity. In fact, this n→π* interaction is either absent or significantly weakened in other organic peroxides, potentially explaining some of the differences in their thermal properties. BPO exhibits a relatively high decomposition temperature (T_0_ = 105 °C) and heat of decomposition (ΔH^d^ = 242 kJ mol^–1^). In contrast, more labile species such as TBHP (*tert*-butyl hydroperoxide, T_0_ = 65 °C, ΔH^d^ = 144 kJ mol^–1^) or LPO (lauroyl peroxide, T_0_ = 70 °C) lack the extended conjugation and stabilizing intramolecular interactions found in BPO, which may contribute to their lower thermal stability.^49^

Taken together, these findings highlight the critical role of subtle intramolecular electronic effects, such as n→π* delocalization, in modulating stability, reactivity, and potentially the hazardous behavior of organic peroxides.

As previously mentioned, several unidentified lines remain present in the experimental spectrum, most likely originating from the photofragmentation process during laser ablation. To elucidate their nature, we consulted molecular spectroscopy databases, including the Cologne Database for Molecular Spectroscopy (CDMS), as well as relevant literature. Through this analysis, we were able to assign several of the observed transitions to several molecular species (see Table S4 for the list of the assigned transitions). The chemical species coming from photofragmentation are illustrated in Figure 4, and in the following we proceed to explain their origin.

**Figure 4:**
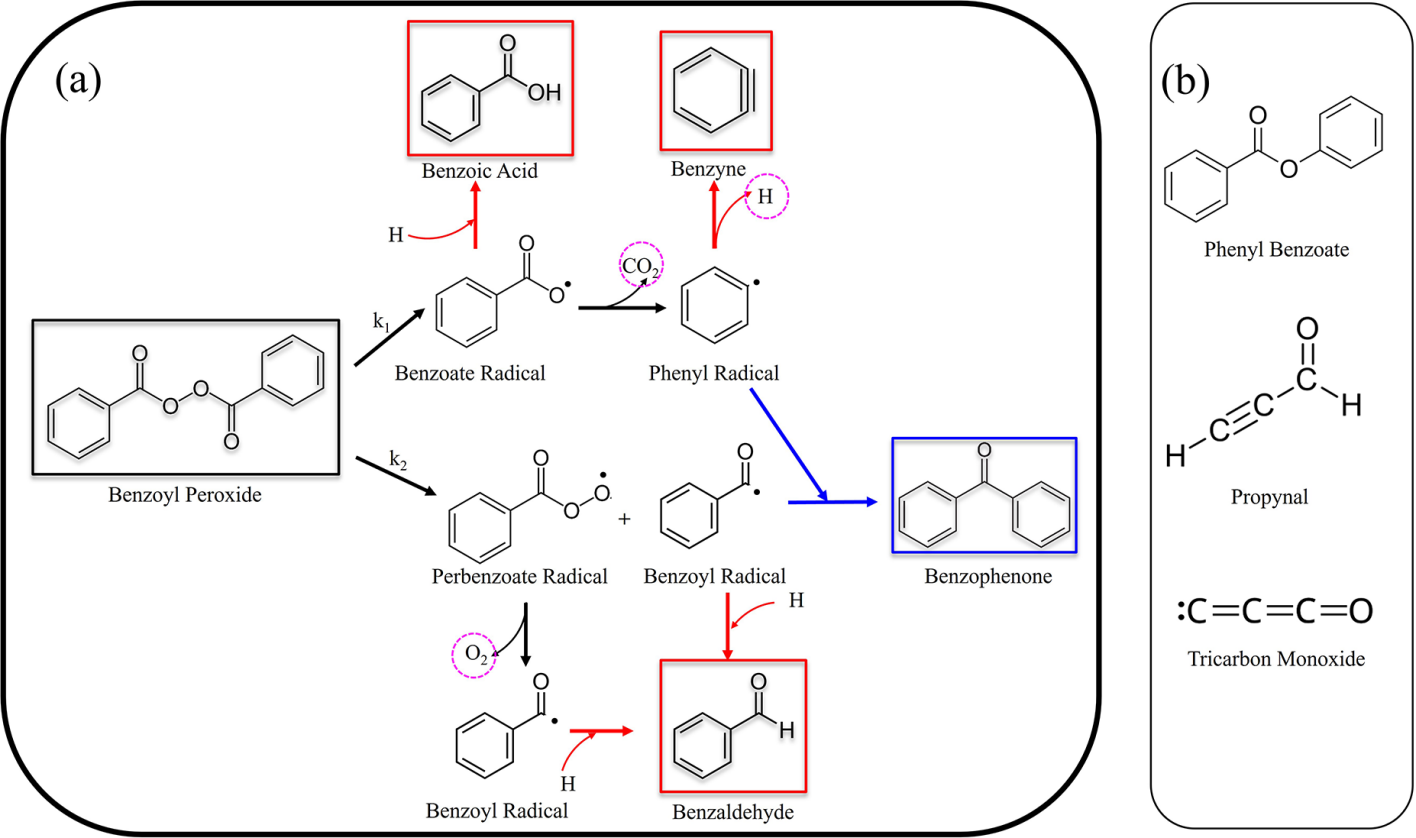
(a) Proposed fragmentation pathway highlighting the detected photofragment species. Framed molecules are those chemical species detected in the rotational spectrum of BPO. (b) Chemical species tentatively detected (few rotational lines) in the rotational spectrum of BPO.

According to established decomposition pathways,^10–14^ benzoyl peroxide undergoes initial homolytic cleavage of the O–O bond, producing two benzoate radicals (see Scheme 1 and Figure 4). These radicals may subsequently undergo decarboxylation to generate phenyl radicals. Despite extensive efforts, we did not detect clear spectroscopic evidence for these radical intermediates in the gas phase probably because they rapidly deactivate or react with hydrogen or another species generated during the plasma or during the cooling in the supersonic expansion. However, we observed strong signatures corresponding to their nonradical forms, i.e., benzoic acid and benzyne. The presence of these species could potentially provide indirect evidence that they are downstream products of this decomposition route, providing as well indirect evidence for the transient presence of reactive intermediates. Moreover, the estimated abundance of benzoic acid is even larger than that of BPO, confirming the large propensity of BPO to undergo initial homolytic cleavage of the O–O bond. Furthermore, QTAIM analysis of BPO (Table S3) supports our observations. The O–O (O14–O15) bond is the weakest, showing the lowest electron density (ρ = 0.294) and only positive Laplacian (Δρ = +0.022), indicating a closed-shell interaction characteristic of a weak and easily cleavable bond. This is further supported by its low |V/G| ratio (−1.97) and the least negative total energy density (H = −0.204), signifying minimal electronic stabilization.

At the same time, the detection of benzaldehyde provides strong indirect evidence for the presence of the benzoyl radical. To our knowledge, the latter has never been detected as BPO decomposition byproduct, although it was suggested that it could be formed by cleavage of the O=C–O bond.^15^ According to the calculations done by the authors, the cleave of this bond requires more energy than the cleave of the O–O bond, but it is second to the main mechanism. Our results provide indirect evidence supporting this secondary route, as well as its lower kinetics, as the estimated abundance of benzaldehyde is considerably lower than that of benzoic acid. Another indirect evidence of this route is the presence of benzophenone, which we believe arises from the bottom-up recombination process involving phenyl and benzoyl radicals, confirming the crossing between these two routes. In fact, the estimated abundance of benzophenone is like that of benzaldehyde, confirming that this new route is less likely than the main route. Moreover, this parallel mechanism also explains the generation of O_2_ in the mass spectrum of the gaseous products of the photolysis of BP conducted by Kuzina et al.^14^

Finally, this new observation prompted us to consider other possible recombination products, such as phenyl benzoate, a species previously reported in related BPO studies.^11–13^ Despite the fact that we could not make a proper fit with enough rotational transitions in the spectrum, we found some rotational transitions that seem to match with the predicted rotational constants (see Tables S5 and S6) of the main conformer.

While the detection of these closed-shell products is consistent with the expected homolytic O–O bond cleavage pathway known from thermal studies, we acknowledge that the laser ablation environment involves complex processes that may contribute to product formation. However, several experimental observations support our interpretation that the detected fragments primarily arise from thermal-like decomposition rather than direct photofragmentation: (i) laser parameters and sample preparation were carefully optimized to minimize photofragmentation effects, as monitored through the absence of common photofragmentation products; (ii) the anomalously high abundance of the detected fragments relative to the parent BPO signal suggests efficient thermal decomposition during the ablation process; and (iii) the observed species correspond exclusively to those predicted by established organic chemistry mechanisms, with no additional photofragmentation byproducts typically observed in our extensive experience with laser ablation studies. The observed species should therefore be considered as strong indirect evidence supporting the involvement of radical intermediates analogous to those in thermal decomposition mechanisms. Nevertheless, we recognize that the plasma environment and photochemical processes inherent to laser ablation may contribute to product formation through pathways that complement purely thermal mechanisms. Therefore, while our observations provide valuable insights into BPO’s fragmentation routes and demonstrate remarkable consistency with established thermal decomposition pathways, the molecular-level understanding gained from this gas-phase investigation should be considered alongside, rather than as a direct replacement for, condensed-phase thermal studies. This work thus bridges gas-phase molecular spectroscopy with condensed-phase reactivity, offering a complementary perspective on the fundamental processes governing peroxide decomposition.

## Supplementary Material



## Notes

Detailed experimental and theoretical section, Cartesian coordinates of BPO, measured frequencies for the detected rotamer of BPO, topological analysis, NCIPlots of the three conformers of PBO, measured frequencies for the detected fragments of BPO, and spectroscopic parameters of phenyl benzoate (PDF)
