# Supplementary material for: Fragmentation Dynamics of Benzoyl Peroxide: Insights from Rotational Spectroscopy
Source: J Phys Chem Lett. 2025 Oct 30;16(44):11597–603. doi: 10.1021/acs.jpclett.5c02600 (PMC12598921; doi:10.1021/acs.jpclett.5c02600)
Supplement: Supplementary file 1 [file jz5c02600_si_001.pdf]

Supporting information for:

Fragmentation Dynamics of Benzoyl Peroxide: Insights from Rotational Spectroscopy

Sergio Mato [a], Sofía Municio [a], José Luis Alonso [a], Elena R. Alonso [a], Iker León [a]*

[a] *Grupo de Espectrocopía Molecular (GEM), Edificio Quifima, Laboratorios de Espectroscopia y Bioespectroscopia, Unidad Asociada CSIC, Parque Científico UVa, Universidad de Valladolid, 47011, Valladolid, Spain*

* E-mail: Iker.leon@uva.es

METHODOLOGY

Experimental

Commercially available wetted benzoyl peroxide (Glentham), a crystalline white solid (m.p 378K), was used directly without any further purification. The sample underwent fine pulverization and was thoroughly mixed with a commercial binder (Peoval). This mixture was subsequently subjected to hydraulic pressing to form a solid rod, which was then positioned within the ablation nozzle. The rotational spectrum was acquired using a broadband chirped-pulse Fourier-transform microwave (CP-FTMW) spectrometer, operating within the 6–14 GHz frequency range.[1–3]

Gas-phase benzoyl peroxide was generated via picosecond Nd:YAG laser ablation (20 ps pulse width, 355 nm) with a fluence of 15 mJ. The resulting vaporized products were diluted in neon at a stagnation pressure of 10 bar. Following vaporization, a pulsed valve, connected to the spectrometer's vacuum chamber and operating at a 2 Hz repetition rate, facilitated a supersonic expansion to achieve molecular cooling. A 24 GS s^{-1} arbitrary waveform generator (AWG) produced a 4 μs fast chirp microwave pulse, which was amplified by a 200 W solid-state amplifier (SSA) to induce macroscopic polarization of the molecules within the spectral region of interest. Up to 95,000 free induction decays (FIDs) with a 10 μs acquisition time were recorded in the time domain, with 4 FIDs averaged per valve cycle. The FIDs were then averaged in the time domain, followed by a fast Fourier transform to obtain the frequency-domain spectrum. The recorded lines have an uncertainty of about 30 kHz.

Computational

A systematic approach was employed to explore the conformational landscape of BPO and possible fragments or complexes. Initially, a comprehensive survey of potential energy minima was conducted using rapid molecular mechanics methods, specifically "Large scales Low Mode" and Monte Carlo-based search algorithms.[4] Each resulting molecular structure was subsequently subjected to geometric re-optimization using density functional theory (DFT) at the B3LYP-GD3BJ level, with the 6-311++G(d,p) basis set.[5–8] The optimized structures were verified as local minima on the potential energy surface (PES) by confirming the absence of imaginary eigenvalues in their Hessian matrices. Zero-point energy (ZPE) corrections were obtained through harmonic frequency calculations.

Figure S1. BPO Atom numbering.

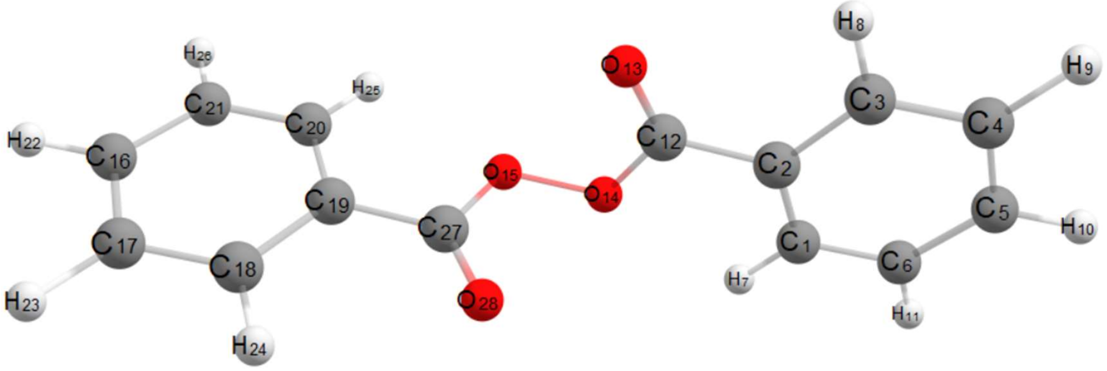

Table S1. Cartesian coordinates in Angstroms of BPO conformers from the optimized B3LYP-D3(BJ)/6-311G++(d,p) levels.

BPO-I				BPO-II				BPO-III			
Atom	X	Y	Z	Atom	X	Y	Z	Atom	X	Y	Z
C1	-3.33	-0.79	0.79	C1	2.58	-0.27	-1.08	C1	-2.19	0.32	-1.20
C2	-2.81	0.23	0.00	C2	1.99	-0.42	0.19	C2	-2.13	-0.40	0.00
C3	-3.66	0.98	-0.83	C3	2.63	0.10	1.32	C3	-3.18	-0.33	0.92
C4	-5.03	0.71	-0.85	C4	3.85	0.75	1.19	C4	-4.29	0.46	0.65
C5	-5.54	-0.31	-0.05	C5	4.44	0.89	-0.07	C5	-4.35	1.19	-0.55
C6	-4.70	-1.06	0.76	C6	3.80	0.38	-1.20	C6	-3.30	1.12	-1.46
H7	-2.67	-1.38	1.42	H7	2.09	-0.68	-1.95	H7	-1.38	0.27	-1.91
H8	-3.24	1.77	-1.44	H8	2.17	-0.03	2.29	H8	-3.11	-0.90	1.84
H9	-5.68	1.29	-1.48	H9	4.35	1.15	2.07	H9	-5.10	0.52	1.36
H10	-6.61	-0.53	-0.07	H10	5.39	1.40	-0.17	H10	-5.21	1.80	-0.76
H11	-5.10	-1.86	1.38	H11	4.26	0.49	-2.18	H11	-3.35	1.68	-2.39
C12	-1.37	0.58	-0.03	C12	0.69	-1.10	0.40	C12	-0.97	-1.26	0.37
O13	-0.84	1.37	-0.76	O13	0.19	-1.37	1.45	O13	-0.83	-1.88	1.38
O14	-0.70	-0.15	0.93	O14	0.09	-1.36	-0.82	O14	-0.03	-1.22	-0.64
O15	0.70	0.15	0.93	O15	-1.10	-2.15	-0.67	O15	1.07	-2.12	-0.38
C16	5.54	0.31	-0.05	C16	-2.35	2.81	-0.05	C16	2.51	2.71	0.58
C17	5.03	-0.71	-0.85	C17	-2.87	2.04	0.99	C17	3.23	2.17	-0.49
C18	3.66	-0.98	-0.83	C18	-2.80	0.65	0.93	C18	3.14	0.81	-0.78
C19	2.81	-0.23	0.00	C19	-2.19	0.03	-0.16	C19	2.30	-0.01	-0.01
C20	3.33	0.79	0.79	C20	-1.68	0.80	-1.21	C20	1.59	0.53	1.07
C21	4.70	1.06	0.76	C21	-1.77	2.19	-1.16	C21	1.71	1.88	1.37
H22	6.61	0.53	-0.07	H22	-2.41	3.89	-0.01	H22	2.59	3.76	0.81
H23	5.68	-1.29	-1.48	H23	-3.32	2.52	1.85	H23	3.86	2.81	-1.10
H24	3.24	-1.77	-1.44	H24	-3.20	0.04	1.73	H24	3.70	0.38	-1.59
H25	2.67	1.38	1.42	H25	-1.22	0.32	-2.06	H25	0.98	-0.11	1.69
H26	5.10	1.86	1.38	H26	-1.38	2.78	-1.97	H26	1.17	2.29	2.21
C27	1.37	-0.58	-0.03	C27	-2.21	-1.45	-0.18	C27	2.29	-1.46	-0.31
O28	0.84	-1.37	-0.76	O28	-3.12	-2.15	0.16	O28	3.25	-2.15	-0.50

Table S2. Measured frequencies and residuals in MHz for the rotational transitions of Rotamer I (BPO-I).

J´	K´$_a$	K´$_c$	J´´	K´´$_a$	K´´$_c$	ν_{obs}	ν_{obs}-ν_{cal}
14	1	13	13	0	13	6175.977	0.013
5	2	3	4	1	3	6261.626	0.012
5	2	4	4	1	4	6274.100	0.033
15	1	14	14	0	14	6511.539	-0.007
6	2	4	5	1	4	6584.515	0.010
6	2	5	5	1	5	6603.189	0.018
16	1	15	15	0	15	6847.802	-0.007
7	2	5	6	1	5	6906.826	0.037
7	2	6	6	1	6	6932.924	0.024
17	1	16	16	0	16	7184.747	-0.013
8	2	6	7	1	6	7228.487	0.017
8	2	7	7	1	7	7263.273	0.021
18	1	17	17	0	17	7522.364	-0.045
9	2	7	8	1	7	7549.569	0.014
9	2	8	8	1	8	7594.259	0.029
18	3	16	18	2	16	7727.271	0.034
17	3	15	17	2	15	7727.778	0.021
16	3	14	16	2	14	7728.193	-0.002
15	3	13	15	2	13	7728.566	0.005
14	3	12	14	2	12	7728.833	-0.029
13	3	11	13	2	11	7729.177	-0.026
12	3	10	12	2	10	7729.177	-0.026
10	3	8	10	2	8	7729.581	0.008
3	3	0	3	2	2	7729.830	-0.010
17	3	14	17	2	16	7730.663	-0.032
19	1	18	18	0	18	7860.712	-0.051
10	2	8	9	1	8	7870.082	0.030
10	2	9	9	1	9	7925.855	0.022
11	2	9	10	1	9	8189.959	-0.008
11	2	10	10	1	10	8258.094	0.033
12	2	10	11	1	10	8509.327	0.015
12	2	11	11	1	11	8590.944	0.028
3	3	0	2	2	0	8707.793	-0.001
3	3	1	2	2	1	8707.793	-0.001
13	2	11	12	1	11	8828.095	0.002
13	2	12	12	1	12	8924.424	0.027
4	3	1	3	2	1	9033.779	0.000
4	3	2	3	2	2	9033.779	0.000
14	2	12	13	1	12	9146.316	-0.007
14	2	13	13	1	13	9258.524	0.018
5	3	2	4	2	2	9359.769	0.006
5	3	3	4	2	3	9359.769	0.006
15	2	13	14	1	13	9464.020	0.007
6	3	3	5	2	3	9685.751	0.005
6	3	4	5	2	4	9685.751	0.005
7	3	4	6	2	4	10011.667	-0.034
7	3	5	6	2	5	10011.833	0.080

17	2	15	16	1	15	10097.778	-0.041
8	3	5	7	2	5	10337.697	-0.009
8	3	6	7	2	6	10337.697	-0.009
9	3	6	8	2	6	10663.620	0.019
9	3	7	8	2	7	10663.751	-0.009
19	2	17	18	1	17	10729.564	-0.056
13	4	9	13	3	11	10821.719	0.004
10	3	7	9	2	7	10989.572	0.046
10	3	8	9	2	8	10989.755	-0.020
11	3	8	10	2	8	11315.434	0.007
11	3	9	10	2	9	11315.791	-0.009
12	3	9	11	2	9	11641.297	-0.004
12	3	10	11	2	10	11641.833	-0.006
4	4	0	3	3	0	12125.657	-0.058
4	4	1	3	3	1	12125.657	-0.058
14	3	11	13	2	11	12292.990	0.051
5	4	1	4	3	1	12451.656	-0.043
5	4	2	4	3	2	12451.656	-0.043
6	4	2	5	3	2	12777.647	-0.038
6	4	3	5	3	3	12777.647	-0.038
7	4	3	6	3	3	13103.631	-0.037
7	4	4	6	3	4	13103.631	-0.037

Table S3. Topological properties at the bond critical points (BCP) for the most stable conformer of BPO calculated using the QTAIM analysis at B3LYP-D3BJ/6-311++G(d,p).

Bond	ρ^a	$\Delta^2\rho^b$	V^c	G^d	ε^e	V/G	H^f
C1 C2	0.306	-0.842	-0.406	0.098	0.193	-4.149	-0.308
C3 C2	0.307	-0.850	-0.409	0.098	0.193	-4.164	-0.311
C2 C12	0.267	-0.679	-0.302	0.066	0.120	-4.562	-0.236
H9 C4	0.283	-0.976	-0.320	0.038	0.017	-8.444	-0.282
C4 C3	0.312	-0.878	-0.423	0.102	0.197	-4.159	-0.321
C5 C6	0.309	-0.868	-0.416	0.099	0.191	-4.185	-0.316
C5 C4	0.309	-0.867	-0.415	0.099	0.190	-4.187	-0.316
H11 C6	0.283	-0.976	-0.320	0.038	0.017	-8.428	-0.282
C6 C1	0.311	-0.874	-0.421	0.101	0.198	-4.159	-0.320
H10 C5	0.283	-0.978	-0.319	0.037	0.013	-8.533	-0.282
C1 H7	0.286	-0.999	-0.322	0.036	0.013	-8.887	-0.286
O14 C12	0.276	-0.521	-0.617	0.243	0.011	-2.535	-0.374
C12 O13	0.426	-0.120	-1.433	0.702	0.082	-2.043	-0.732
C3 H8	0.285	-0.995	-0.320	0.036	0.013	-8.993	-0.284
O14 O15	0.294	0.022	-0.414	0.210	0.011	-1.973	-0.204
C27 C19	0.267	-0.679	-0.302	0.066	0.120	-4.562	-0.236
C17 C16	0.309	-0.867	-0.415	0.099	0.190	-4.187	-0.316
C19 C18	0.307	-0.850	-0.409	0.098	0.193	-4.164	-0.311
C17 H23	0.283	-0.976	-0.320	0.038	0.017	-8.444	-0.282
C18 C17	0.312	-0.878	-0.423	0.102	0.197	-4.159	-0.321
O15 C27	0.276	-0.521	-0.617	0.243	0.011	-2.535	-0.374
C21 C16	0.309	-0.868	-0.416	0.099	0.191	-4.185	-0.316
C19 C20	0.306	-0.842	-0.406	0.098	0.193	-4.149	-0.308
H25 C20	0.286	-0.999	-0.322	0.036	0.013	-8.887	-0.286
C16 H22	0.283	-0.978	-0.319	0.037	0.013	-8.533	-0.282
C21 H26	0.283	-0.976	-0.320	0.038	0.017	-8.428	-0.282
C20 C21	0.311	-0.874	-0.421	0.101	0.198	-4.159	-0.320
H24 C18	0.285	-0.995	-0.320	0.036	0.013	-8.993	-0.284
O28 C27	0.426	-0.120	-1.433	0.702	0.082	-2.043	-0.732

[a] Electron density at the BCP. [b] Laplacian at the BCP (au). [c] Potential energy density (au). [d] Gradient kinetic energy density (au). [e] Bond ellipticity. [f] Total electronic energy density (au).

Figure S2. NCIplot representation of non-covalent interactions for the three conformers of BPO. Red surfaces correspond to strong repulsion forces, blue to strong attraction forces and green surfaces to weak attractive interactions. An isovalue of 0.35 a.u was used.

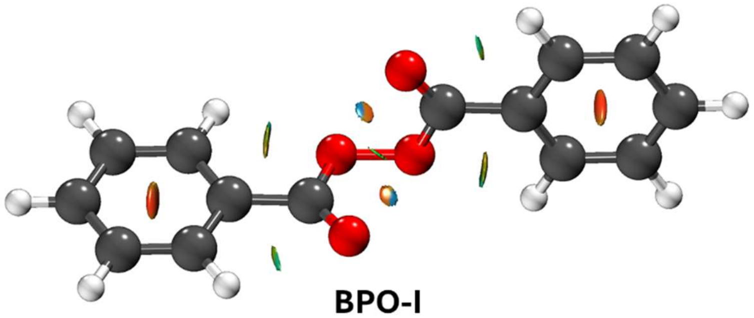

BPO-I

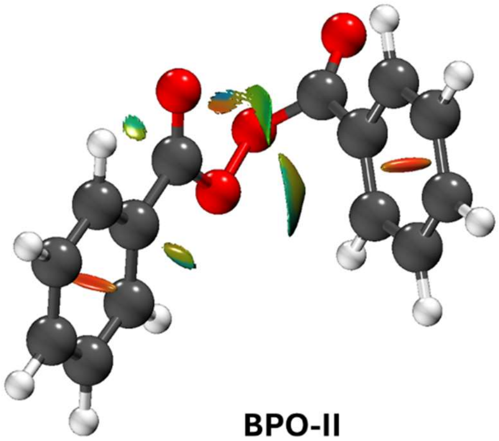

BPO-II

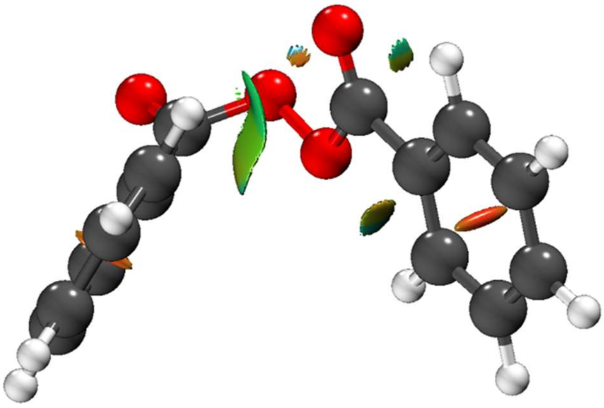

BPO-III

Table S4. Measured frequencies and residuals in MHz for the rotational transitions of the detected photofragments.

Benzoic Acid (PhCOOH): 49 Lines						
J´	K´a	K´c	J´´	K´´a	K´´c	ν_{obs}
3	1	3	2	1	2	6023.494
3	0	3	2	0	2	6387.443
4	0	4	3	1	3	6405.364
3	2	2	2	2	1	6479.633
3	2	1	2	2	0	6571.827
2	1	2	1	0	1	6669.975
3	1	2	2	1	1	6906.524
6	1	5	6	0	6	6947.326
6	2	4	6	1	5	6964.364
5	2	3	5	1	4	7043.428
7	2	5	7	1	6	7122.889
4	2	2	4	1	3	7290.520
6	1	5	5	2	4	7347.763
8	2	6	8	1	7	7573.480
3	2	1	3	1	2	7623.415
2	2	0	2	1	1	7958.168
4	1	4	3	1	3	8006.710
3	1	3	2	0	2	8396.992
4	0	4	3	0	3	8414.916
7	1	6	7	0	7	8597.137
4	2	3	3	2	2	8621.373
4	3	2	3	3	1	8682.814
4	3	1	3	3	0	8689.717
5	0	5	4	1	4	8770.603
2	2	1	2	1	2	8819.124
4	2	2	3	2	1	8845.916
4	1	3	3	1	2	9178.809
3	2	2	3	1	3	9275.268
4	2	3	4	1	4	9889.930
5	1	5	4	1	4	9972.011
4	1	4	3	0	3	10016.266
7	1	6	6	2	5	10265.210
5	0	5	4	0	4	10371.947
8	1	7	8	0	8	10490.187
5	2	4	5	1	5	10665.574
5	2	4	4	2	3	10747.665
10	3	7	10	2	8	10813.231
5	3	3	4	3	2	10867.982
5	3	2	4	3	1	10891.922
6	0	6	5	1	5	11066.002
5	2	3	4	2	2	11174.720
5	1	4	4	1	3	11421.815
5	1	5	4	0	4	11573.362

6	2	5	6	1	6	11602.058
6	1	6	5	1	5	11918.448
6	0	6	5	0	5	12267.432
2	2	1	1	1	0	12549.399
2	2	0	1	1	1	12867.429
7	0	7	6	1	6	13270.129
Benzaldehyde (PhCHO): 9 Lines						
J´	K'_a	K'_c	J´´	K''_a	K''_c	ν_{obs}
3	0	3	2	0	2	8207.067
3	2	2	2	2	1	8306.850
3	1	2	2	1	1	8829.937
4	1	4	3	1	3	10309.474
4	0	4	3	0	3	10831.402
4	2	3	3	2	2	11056.274
4	2	2	3	2	1	11300.565
4	1	3	3	1	2	11741.242
5	1	5	4	1	4	12846.659
Benzophenone (PhCOPh): 14 Lines						
J´	K'_a	K'_c	J´´	K''_a	K''_c	ν_{obs}
8	1	8	7	0	7	6537.248
6	2	5	5	1	4	8086.838
13	1	12	12	2	11	8189.959
7	2	6	6	1	5	8678.683
3	3	1	2	2	0	8846.890
3	3	0	2	2	1	8848.910
6	2	4	5	1	5	9102.451
8	2	7	7	1	6	9244.091
4	3	2	3	2	1	9609.379
5	3	3	4	2	2	10364.165
5	3	2	4	2	3	10394.815
6	3	4	5	2	3	11105.949
4	4	1	3	3	0	12233.697
4	4	0	3	3	1	12233.697
Benzyne (B): 3 Lines						
J´	K'_a	K'_c	J´´	K''_a	K''_c	ν_{obs}
3	2	1	3	1	2	7753.397
2	1	1	2	0	2	8073.193
1	1	1	0	0	0	10130.060
Propynal: 1 Line						
J´	K'_a	K'_c	J´´	K''_a	K''_c	ν_{obs}
1	0	1	0	0	0	9325.812
C_3O: 1 Line						
J´	K'_a	K'_c	J´´	K''_a	K''_c	ν_{obs}
1	0	0	0	0	0	9621.767

Table S5. Measured frequencies and residuals in MHz for the rotational transitions of the tentative phenyl benzoate conformer.

J´	K´a	K´c	J´´	K´´a	K´´c	ν_{obs}	ν_{obs}-ν_{cal}
3	3	1	2	2	0	11124.123	-0.013
3	3	0	2	2	1	11124.123	-0.013
4	3	1	3	2	2	11641.833	0.011
4	3	2	3	2	1	11641.297	0.003
9	2	7	8	1	8	10729.564	-0.001
9	1	9	8	0	8	6216.658	-0.001

Table S6. Spectroscopic parameters for the two conformers of phenyl benzoate at B3LYP-GD3BJ/6-311++(d,p) level and tentative experimental fitting.

Constants[a]	Experimetal	PhCOOPh-I	PhCOOPh-II
A[a]	2173.08233(108)	2127	1007
B	266.90305(46)	277	457
C	250.51922(43)	260	375
$\|\mu_a\|$	No	-1.0	-0.3
$\|\mu_b\|$	Yes	-1.6	-4.6
$\|\mu_c\|$	No	0.6	-0.1
ΔE		0	1958
ΔE_{ZPE}		0	1924
ΔG		0	2120
N	6		
σ	7.7		

[a]*A*, *B*, and *C* represent the rotational constants (in MHz); μ_a, μ_b and μ_c are the electric dipole moment components (in D); N is the number of lines included in the fit; σ is the rms error of the fit (in kHz); ΔE is the relative electronic energy (in cm^{-1}). ΔE_{ZPE} is the relative energies (in cm^{-1}) with respect to the global minimum, considering the zero-point energy (ZPE). ΔG is the Gibbs free energies (in cm^{-1}, p = 1 bar and T = 298 K).

REFERENCES

[1] R. Aguado, S. Mata, M. Sanz-Novo, E. R. Alonso, I. León, J. L. Alonso, *J. Phys. Chem. Lett.* **2022**, *13*, 9991–9996.

[2] S. Mato, S. Municio, J. L. Alonso, E. R. Alonso, I. León, *ChemistryOpen* **2025**, e202400490.

[3] M. Sanz-Novo, M. Mato, I. León, A. M. Echavarren, J. L. Alonso, *Angew. Chemie - Int. Ed.* **2022**, *61*, 1–6.

[4] 2018. Schrödinger Release 2018-3: Maestro Schrödinger, LLC, New York, NY, **n.d.**

[5] A. D. Becke, *J. Chem. Phys.* **1993**, *98*, 1372–1377.

[6] T. Schwabe, S. Grimme, T. Schwabe, S. Grimme, *PCCP* **2007**, *9*, 3397.

[7] S. Grimme, J. Antony, S. Ehrlich, H. Krieg, *J. Chem. Phys.* **2010**, *132*, 154104.

[8] M. J. Frisch, J. A. Pople, J. S. Binkley, *J. Chem. Phys.* **1984**, *80*, 3265–3269.
